# Gut microbiota–bile acid‐vitamin D axis plays an important role in determining oocyte quality and embryonic development

**DOI:** 10.1002/ctm2.1236

**Published:** 2023-10-17

**Authors:** Ang Li, Fei Li, Wei Song, Zi‐Li Lei, Qian‐Qian Sha, Shao‐Yuan Liu, Chang‐Yin Zhou, Xue Zhang, Xiao‐Zhen Li, Heide Schatten, Teng Zhang, Qing‐Yuan Sun, Xiang‐Hong Ou

**Affiliations:** ^1^ Fertility Preservation Lab Guangdong‐Hong Kong Metabolism and Reproduction Joint Laboratory Reproductive Medicine Center Guangdong Second Provincial General Hospital Guangzhou China; ^2^ State Key Laboratory of Reproductive Regulation and Breeding of Grassland Livestock College of Life Sciences Inner Mongolia University Hohhot China; ^3^ Guangdong Metabolic Diseases Research Center of Integrated Chinese and Western Medicine Key Laboratory of Glucolipid Metabolic Disorder Ministry of Education of China Institute of Chinese Medicine Guangdong Traditional Chinese Medicine (TCM) Key Laboratory for Metabolic Diseases Guangdong Pharmaceutical University Guangzhou China; ^4^ Department of Veterinary Pathobiology University of Missouri‐Columbia Columbia Missouri USA

## Abstract

**Objective:**

To reveal whether gut microbiota and their metabolites are correlated with oocyte quality decline caused by circadian rhythm disruption, and to search possible approaches for improving oocyte quality.

**Design:**

A mouse model exposed to continuous light was established. The oocyte quality, embryonic development, microbial metabolites and gut microbiota were analyzed. Intragastric administration of microbial metabolites was conducted to confirm the relationship between gut microbiota and oocyte quality and embryonic development.

**Results:**

Firstly, we found that oocyte quality and embryonic development decreased in mice exposed to continuous light. Through metabolomics profiling and 16S rDNA‐seq, we found that the intestinal absorption capacity of vitamin D was decreased due to significant decrease of bile acids such as lithocholic acid (LCA), which was significantly associated with increased abundance of *Turicibacter*. Subsequently, the concentrations of anti‐Mullerian hormone (AMH) hormone in blood and melatonin in follicular fluid were reduced, which is the main reason for the decline of oocyte quality and early embryonic development, and this was rescued by injection of vitamin D3 (VD3). Secondly, melatonin rescued oocyte quality and embryonic development by increasing the concentration of lithocholic acid and reducing the concentration of oxidative stress metabolites in the intestine. Thirdly, we found six metabolites that could rescue oocyte quality and early embryonic development, among which LCA of 30 mg/kg and NorDCA of 15 mg/kg had the best rescue effect.

**Conclusion:**

These findings confirm the link between ovarian function and gut microbiota regulation by microbial metabolites and have potential value for improving ovary function.

## Graphical abstract

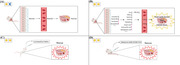

## 1 INTRODUCTION

The physiological functions of most organisms in nature show an alternate rhythm of day and night, which is called circadian rhythm. Circadian rhythm affects all life processes. Daily physiological and behavioral changes are controlled by a highly complex system that includes the main circadian clock of the hypothalamic suprachiasmatic nucleus (SCN), the brain clock outside the SCN and the peripheral oscillator. SCN, the center of the biological clock system, regulates the circadian rhythm of mammals. The light signal reaches the SCN through the retina and then passes on to the pineal gland in the brain. Melatonin is released by the pineal gland at night but its secretion is blocked during the day. Melatonin itself can be fed back to the SCN, which affects the circadian rhythm.^1^ With the development of industrialization and digitalization of modern society, the rhythm of people's life is disrupted, and the competitive pressure of work and study also decreases people's normal sleep time, which leads to an increase in the number of people staying up late, affects the normal secretion of melatonin, and causes circadian rhythm disorder.^2^ Circadian rhythm disorders are regarded to be associated with reproductive system disorders or diseases, cardiovascular diseases, metabolic syndrome, gut microbiota disorders and increased cancer.^3^, ^4^, ^5^

Melatonin is an important hormone needed to regulate circadian rhythm and cope with oxidative stress. Its depletion or lack will clearly lead to circadian rhythm disorders, oxidative stress and other problems.^6^, ^7^ It is believed that melatonin is the bridge between the ‘biological clock system and reproductive system’, which may promote mammalian oocyte maturation, follicular development and embryonic development. In fact, melatonin has been found in human follicular fluid, possibly from ovarian granulosa cells. It is necessary to maintain a high concentration of melatonin in large follicles before ovulation to protect oocytes from oxidative stress associated with ovulation, a process known to produce oxygen free radicals.^7^, ^8^, ^9^ Recently, studies have shown that melatonin supplementation may improve the balance between host and intestinal flora, such as enhancing the stability of the intestinal barrier and microflora regulation ability under sleep deprivation.^10^ Yin et al. reported that melatonin also showed probiotic ability by reversing the imbalance of gut microbiota induced by high fat diet (HFD), thus increasing the abundance of *Bacteroides* and *Alistipes*. In addition, melatonin supplementation improves the production of acetic acid.^11^

One of the important metabolites of gut microbiota is secondary bile acid. Bile acid is derived from cholesterol, which is mainly synthesized by the liver, transported to the intestinal tract and then chemically modified twice under the action of intestinal flora to form a more lipophilic secondary bile acid. Most of the bile acid is absorbed by the liver through enterohepatic circulation, and a small amount of bile acid is excreted through feces.^12^ Bile acid metabolism plays an important regulatory role in many diseases. The latest research by us showed that in MetS sheep induced by high energy diet, the gut microbiota was out of balance, and the levels of many kinds of bile acids (CA, LCA and TUDCA) were decreased, which affected the absorption of vitamin A and damaged spermatogenesis.^13^ Qi et al. revealed that gut microbiota disorder was an important risk factor for polycystic ovary syndrome (PCOS), and bile acid supplementation or increased IL‐22 could improve PCOS.^14^ However, continuous light could lead to the disturbance of oocyte maturation and the decline of oocyte quality, but the underlying mechanism is not clear.^15^

In this study, we first clarified the decline of oocyte quality and embryonic development as well as changes in gut microbiota and their metabolites in mice exposed to continuous light, and then showed that melatonin, secondary bile acids and vitamin D effectively rescued the decline of oocyte quality and embryonic development.

## 2 RESULTS

### 2.1 Continuous light exposure leads to decrease of oocyte quality and reproductive performance

**FIGURE 1:**
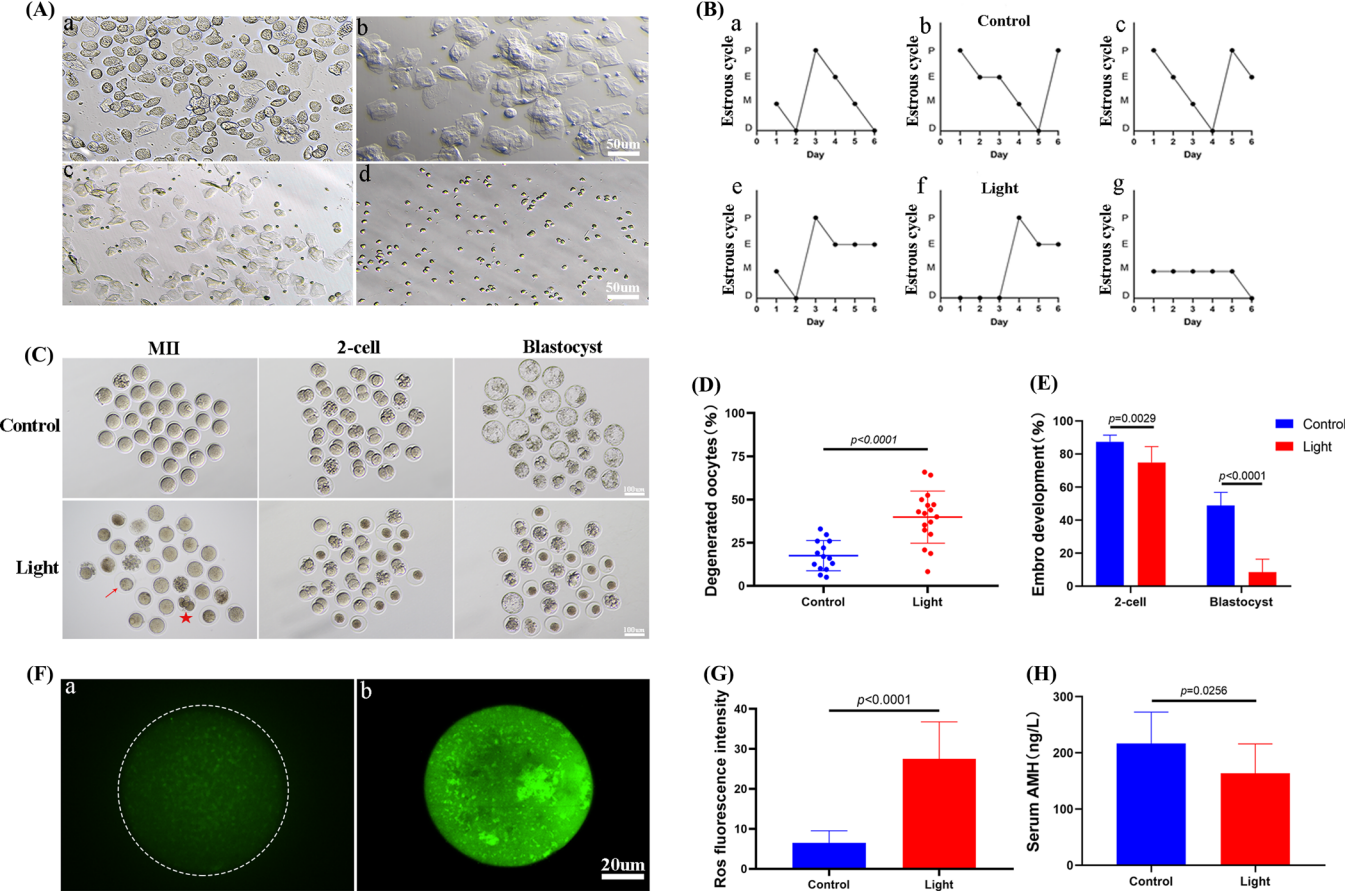
Continuous light exposure leads to the decrease of oocyte quality. (A) [a] Proestrus: vaginal smears showed large round nucleated cells; [b] estrus: vaginal smears mainly showed flat bark‐like irregular keratinized cells; [c] metestrus: vaginal smears began to show small and round white blood cells and keratinized cells; [d] diestrus: vaginal smears mainly showed small and round white blood cells. (B) Representative estrous cycles of two groups. The upper and lower panels represent the control and light groups, respectively. (C) Representative images of the oocytes, two‐cell embryos and blastocyst development in the two groups. (D) Showing degenerated oocyte rates in the two groups. (E) The difference in the two‐cell embryos and blastocyst development were observed in three groups of oocytes. When counting the rate of two‐cell embryos, the denominator was the number of double pronuclear eggs. When counting the rate of blastocyst development, the denominator was the number of two‐cell embryos. (F) Confocal microscopy showing the changes of reactive oxygen species (ROS) production, measured as 2′,7′‐Dichlorodihydrofluorescein diacetate (DCFH) fluorescence. [a], [b] The typical image of the Control and Light groups, respectively. (G) ROS production measured by DCFH fluorescence in the two groups of oocytes. (H) Serum anti‐Mullerian hormone (AMH) concentration.

We established a mouse model exposed to continuous light 24 h a day for 2 months. Compared with the normal light group, the estrous cycle of mice exposed to continuous light was significantly prolonged (Figure 1A,B). As shown in Figure 1C,D, a significant increase in the percentage of degenerated oocytes was observed in the light‐exposed group (referred to as light group thereafter) (Control:17.5% vs. light:39.88%, *p* < .0001). Meanwhile, significantly decreased development to two‐cell embryos and blastocysts was revealed after parthenogenetic activation of oocytes derived from light‐exposed mice (control:87.4% vs. light:75.07%, *p* < .01; control:48.9% vs. light:8.46%, *p* < .0001, Figure 1E,). Then, we determined reactive oxygen species (ROS) production in the two groups of oocytes. ROS distribution in the light‐exposed group was aggregated into clumps in the cytoplasm (Figure 1Fb). The 2′,7′‐Dichlorodihydrofluorescein diacetate (DCFH‐DA) fluorescence intensity was significantly higher in the Light group than that in the control group, indicating an increased production of ROS (*p* < .0001, Figure 1G). To explore the influence of continuous light exposure on reproductive hormones, we measured the concentrations of anti‐Mullerian hormone (AMH) in the two groups, and showed that the level of serum AMH decreased significantly after continuous light exposure (Figure 1H).

### 2.2 Melatonin supplementation rescues the declining quality of oocytes

**FIGURE 2:**
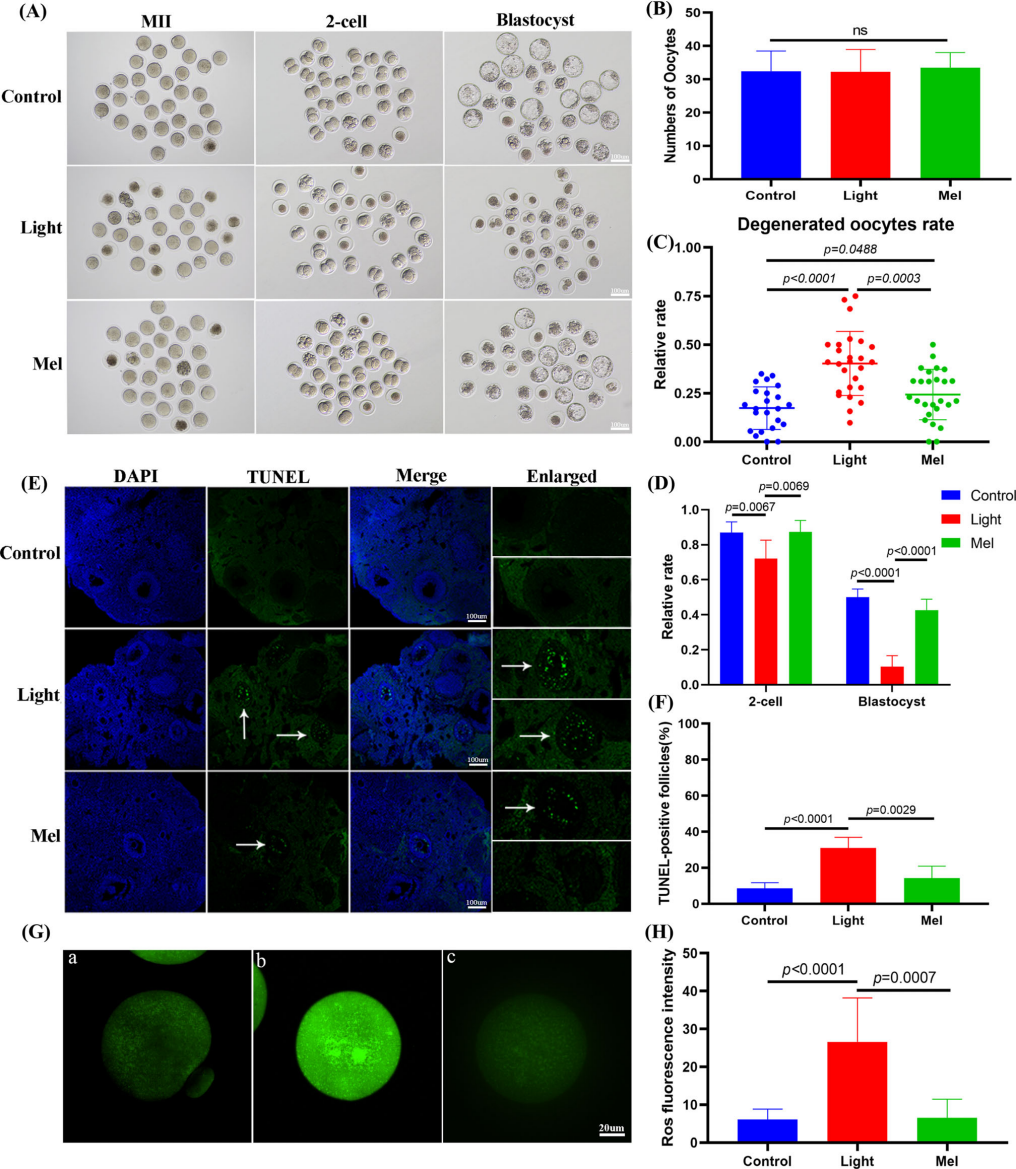
Melatonin supplementation improved oocyte quality and embryonic development, and decreased the percentage of apoptotic follicles and oocyte reactive oxygen species (ROS) level. (A) Representative images of the oocytes, two‐cell embryos and blastocyst development in three groups. (B) Showing the number of superovulated oocytes in the three groups. (D) Terminal ‐deoxynucleotidyl transferase mediated nick end labeling (TUNEL)staining of the ovaries in the three groups. (E) The difference in the two‐cell embryos and blastocysts were observed in three groups. When counting the rate of two‐cell embryos, the denominator was the number of double pronuclear eggs. When counting the rate of blastocysts, the denominator was the number of two‐cell embryos. Data from more than 30 oocytes were analyzed for each group. (F) Quantitative analyses of the number of TUNEL‐positive follicles in the three groups. (G) Confocal microscopy showing the changes in ROS production, measured as 2′,7′‐Dichlorodihydrofluorescein diacetate (DCFH) fluorescence. [a], [b], [c] The typical image of the control, light and Mel groups, respectively. (H) ROS production measured by DCFH fluorescence in the oocytes of the three groups.

In order to explore whether melatonin could improve oocyte quality and embryonic developmental potential under continuous light exposure, we established a rescue model of intraperitoneal injection of 5 mg/kg melatonin every day for 2 months, which was recorded as Mel group. There was no difference in the number of ovulated oocytes among the three groups. However, melatonin supplementation could significantly reduce the rate of degenerated oocytes (control:17.3%, light:40.3%, Mel:24.3%, *p* < .0001) and significantly increase the rate of two‐cell embryos and blastocyst development (control:87%, light:72.1%, Mel:87.3%, *p* < .01; control:50%, light:10.4%, Mel:42.6%, *p* < .0001, Figure 2A–D). Continuous light exposure increased the number of terminal‐deoxynucleotidyl transferase mediated nick end labeling (TUNEL) positive follicles, while melatonin supplementation could significantly reduce this increasing trend (Figure 2E,F). We then determined ROS production in the three groups of oocytes. The DCFH‐DA fluorescence intensity of the Light group was significantly higher than that of the other two groups, indicating that melatonin supplementation could reduce the level of ROS (Figure 2G,H).

**FIGURE 3:**
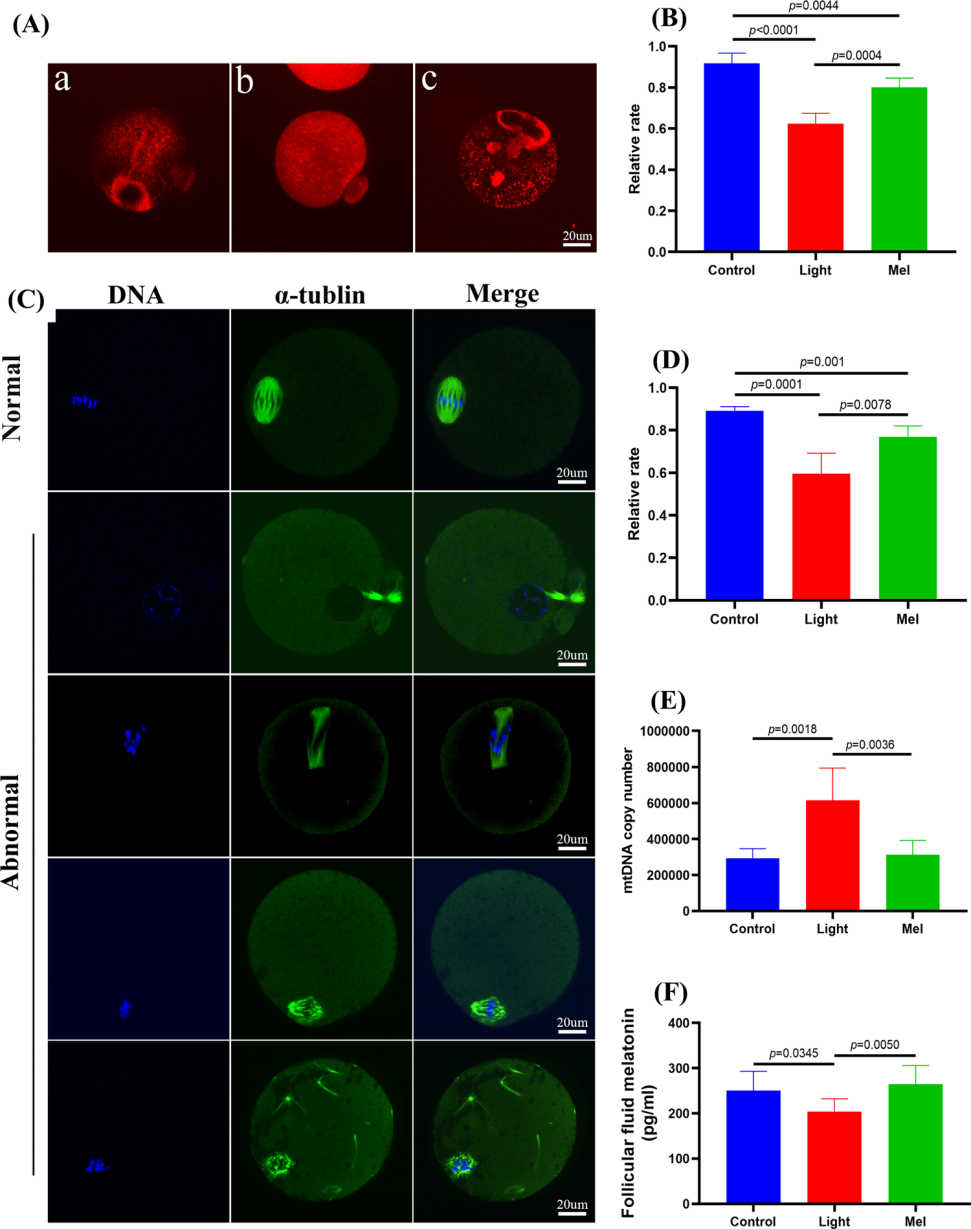
Melatonin supplementation improved oocyte spindle assembly and mitochondrial function, and restored the concentration of melatonin in follicular fluid. (A) Mitochondrial distribution patterns in metaphase II (MII) oocytes were detected by using MitoTracker Red fluorescence staining: [a] polarized distribution [b] homogeneous distribution [i] clustering distribution. (B) The rates of oocytes with normal mitochondrial distribution in the three groups. (C) Showing typical normal and abnormal spindles and chromosomes, respectively. Meiotic spindles in oocytes were stained with α‐tubulin (green) and chromosomes were stained with Hoechst 33342 (blue). (D) The rates of oocytes with normal spindles in the three groups. (E) Mitochondrial DNA (mtDNA) copy number in oocytes as revealed by real‐time PCR analysis. (F) The concentration of melatonin in follicular fluid of the three groups.

Three kinds of mitochondrial distribution patterns were observed in metaphase II (MII) oocytes. Normal mitochondrial distribution showed either polarized distribution or homogeneous distribution, while abnormal mitochondria distribution showed asymmetric clustered distribution (Figure 3A). As shown in Figure 3B, approximately 91.8% and 80.0% of the oocytes in the control group and Mel group showed normal mitochondrial distribution, while the normal mitochondrial distribution rate of oocytes in the light group was only 62.4%. The results showed that the control group oocytes displayed bipolar spindles with focused poles. Various formations of severely abnormal spindles were found in the Light group oocytes, including elongated spindles and spindles with abnormal poles, with the major spindle defect being spindles with no apparent poles (Figure 3C). We found that the normal spindle rate in the Light group was significantly lower than that in the Control group and Mel group (*p* < .01, Figure 3D). We compared the mtDNA copy numbers of the three groups by qPCR. The copy number of mitochondrial DNA in the Light group was significantly higher than that in the other two groups probably due to the compensatory effect (control: 292887, light: 614728, Mel: 312143, *p* < .01, Figure 3E). All these results showed the rescue effects of melatonin on oocyte quality, since injection of melatonin in vivo restored the level of melatonin in the follicular fluid of mice exposed to continuous light (Figure 3F).

### 2.3 The absorption of vitamin D is disrupted due to a drastic reduction in levels of bile acids in the continuous light‐exposed mice

**FIGURE 4:**
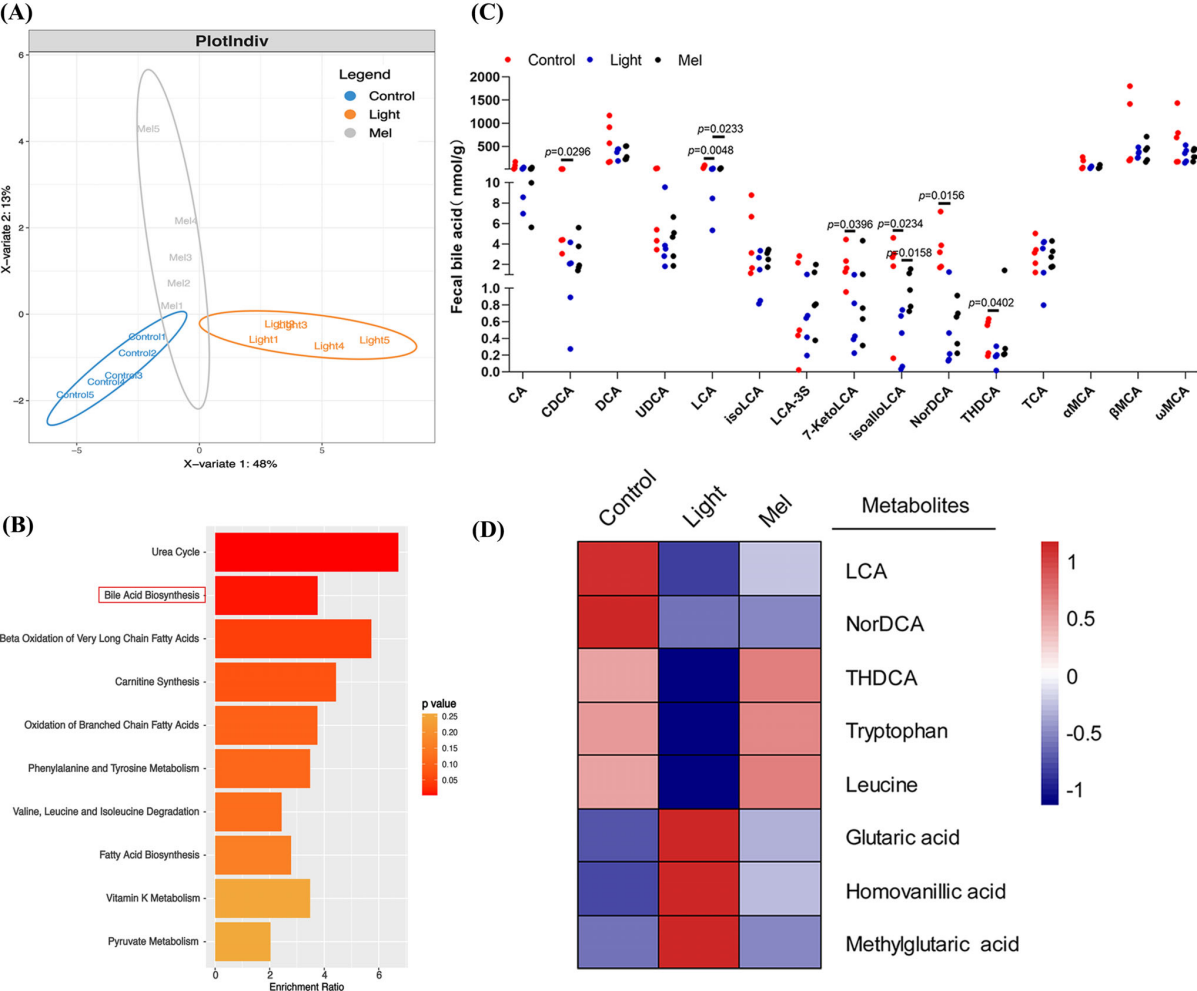
Abnormal bile acid biosynthesis metabolism in mice exposed to continuous light. (A) Orthogonal projections to latent structures‐discriminant analysis (PLS‐DA) score plot for discriminating the microbial metabolites from the Control, Light and Mel groups (*n* = 5). (B) Disturbed metabolic pathways in the Control versus Light groups. (C) The concentrations of fifteen bile acids in the three groups. (D) Heatmaps of the differential metabolites that were altered in the Light and Mel groups compared with the control group.

**FIGURE 5:**
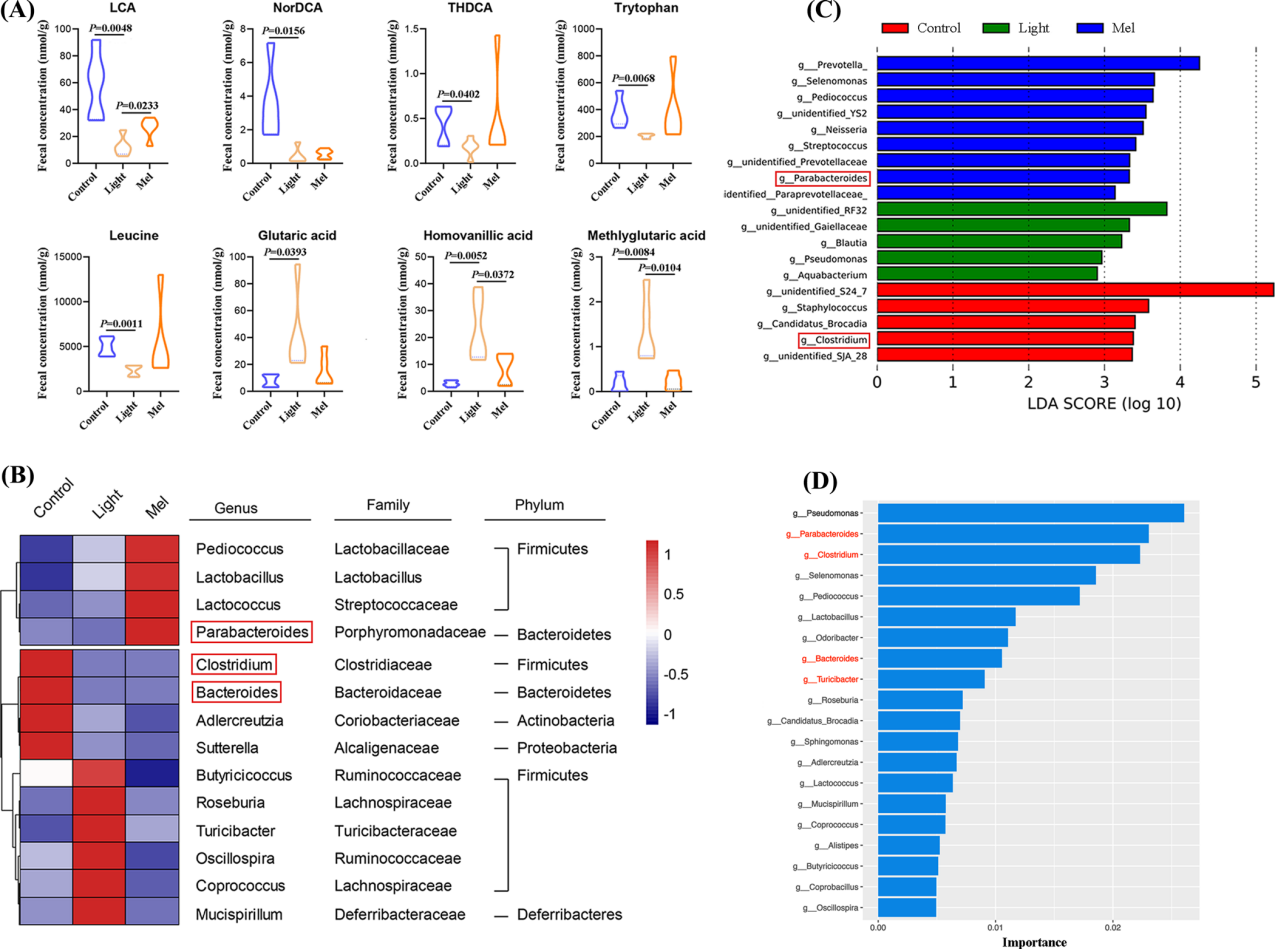
Mice exposed to continuous light suffered gut microbiota dysbiosis. (A) Comparison of the concentrations of lithocholic acid (LCA), NorDCA, THDCA, tryptophan, leucine, glutaric acid, homovanillic acid and methylglutaric acid in the three groups. (B) Heatmap of differential abundance from the light and Mel groups compared with the control group. (C) The LEfSe analysis showing characteristic bacteria in the three groups. (D) Random forests analysis showing 20 important markers of intergroup differences.

In order to clarify whether the decline of oocyte quality was related to microbial metabolites, we used the Ultra Performance Liquid Chromatography Tandem Mass Spectrometry (UPLC‐MS/MS) system to quantitatively detect microbial metabolites in feces. First, using partial least squares discriminant analysis, we found that there was a significant difference between the control group and light group, and the Mel group was more similar to the Control group (Figure 4A). Then, we analyzed the differences of differential metabolites and found that they were mainly concentrated in the pathway of bile acid biosynthesis (Figure 4B). For this reason, we analyzed the qualitative and quantitative results of bile acids, and finally found that the concentrations of many kinds of bile acids such as LCA, NorDCA, THDCA, isoalloLCA decreased significantly in the light group. The concentrations of LCA and isoalloLCA increased significantly after melatonin supplementation (*p* < .05, Figure 4C). Based on the above results, we speculated that LCA plays an important role in improving oocyte quality. LCA is an agonist of vitamin D receptor, and vitamin D plays an important role in female reproduction. In order to critically investigate the mechanism of oocyte quality decline, we detected the concentration of vitamin D in feces and blood and found that it decreased in the Light group (*p* < .001, online figure S1A,B). Further analysis of the data showed that continuous light exposure could also decrease the concentrations of tryptophan and leucine, and increase the concentrations of oxidative stress metabolites, such as glutaric acid, homovanillic acid, methylglutaric acid (Figure 4D). After melatonin supplementation, the concentrations of two essential amino acids increased and the oxidative stress metabolites decreased significantly. The quantitative results of these five metabolites are shown in Figure 5A (*p* < .05). In order to further verify the reliability of our metabolomics profiling, we detected the concentration of tryptophan rate‐limiting enzyme IDO1 by the ELISA method and found that it did increase in the Light group, which verified the decrease of tryptophan concentration (*p* < .0001, online figure S2A,B).

### 2.4 Gut microbiota dysbiosis is induced in the continuous light‐exposed mice

**FIGURE 6:**
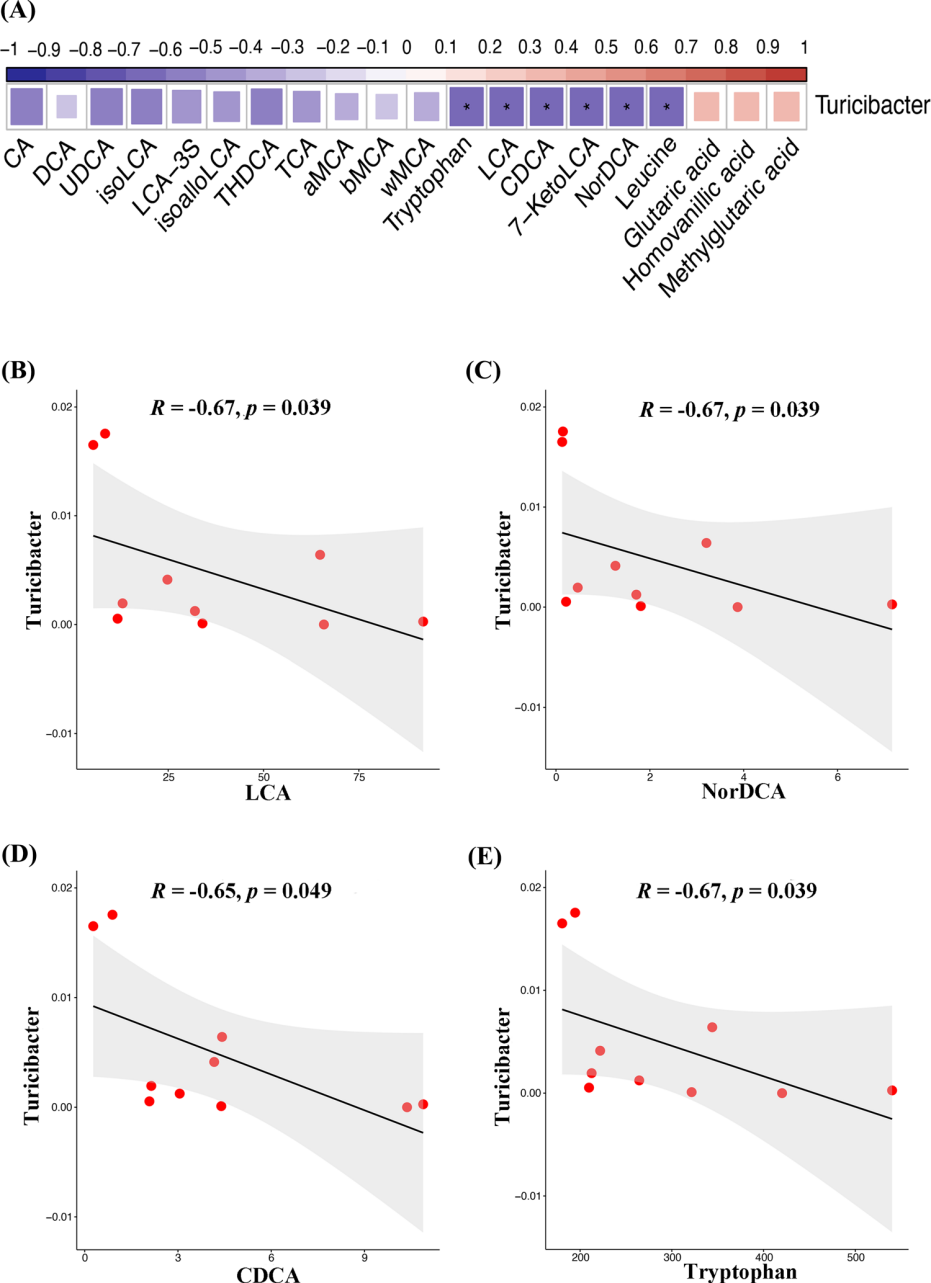
The alterations of gut microbiota were closely associated with the bile acid levels. (A) Spearman's correlation analysis of the *Turicibacter* abundance with differential microbial metabolites levels. (B) Spearman's correlation analysis of *Turicibacter* abundance with lithocholic acid (LCA). (C) Spearman's correlation analysis of *Turicibacter* abundance with NorDCA. (D) Spearman's correlation analysis of *Turicibacter* abundance with CDCA. (E) Spearman's correlation analysis of *Turicibacter* abundance with Tryptophan.

The compositions of gut microbiota play an important role in the metabolic activity of the host. Therefore, we detected the general profiles of gut microbiota by 16SrDNA sequencing of feces in all three groups. At the genus level, we found that the abundance of two kinds of bacteria, namely *Clostridium* and *Bacteroides*, that are involved in the formation of secondary bile acid decreased in the Light group. In addition, the abundance of two bacteria (*Turicibacter* and *Mucispirillum*) causing oxidative stress increased in the Light group. Melatonin supplementation increased the abundance of three probiotics, namely, *Pediococcus*, *Lactobacillus* and *Lactococcus*. In particular, melatonin increased the abundance of *Parabacteroides*, which was involved in the formation of secondary bile acids. At the same time, melatonin supplementation also reduced the increased bacteria abundance caused by continuous light exposure, such as *Turicibacter*, *Mucispirillum*, *Coprococcus* (Figure 5B). In order to find out the characteristic bacteria in different groups, LEfSe was used in this study. At the genus level, the LEfSe analysis showed that the characteristic bacteria of the Mel group included *Parabacteroides*, and the characteristic bacteria of the Control group included *Clostridium* (Figure 5C). Random forests analysis showed 20 important markers of intergroup differences, including *Clostridium*, *Bacteroides*, *Parabacteroides*, *Turicibacter* and *Mucispirillum* (Figure 5D). We focused on the differences in the relative abundance of bacteria among the three groups (online Figure S3A–L). Spearman's correlation analysis was performed. As shown in Figure 6A, we found that *Turicibacter* was negatively correlated with a variety of bile acids and two essential amino acids, and positively correlated with three oxidative stress metabolites (*p* < .05, Figure 6B–E). In addition, we found that *Sphingomonas* was positively correlated with glutaric acid, homovanillic acid, methylglutaric acid and lactic acid (*p* < .05, online Figure S4A–G).

### 2.5 Two secondary bile acids, LCA and NorDCA, have a significant rescue effect on the decline of oocyte quality and embryonic development caused by continuous light exposure

**FIGURE 7:**
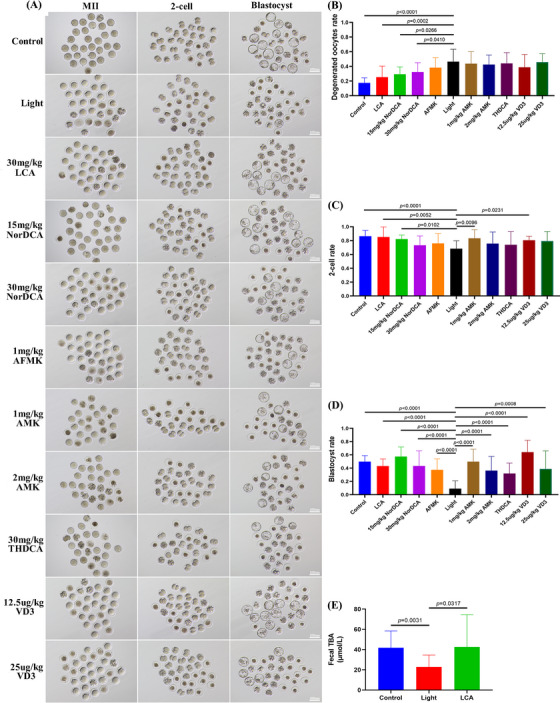
Six metabolites could rescue the oocyte quality and embryonic developmental ability at different degrees in mice exposed to continuous light. (A) Representative images of the oocytes, two‐cell embryos and blastocyst development in the eleven groups. (B) Degenerated oocyte rates in the eleven groups. (C) Two‐cell embryo rates in the eleven groups. (D) Blastocyst development rates in the eleven groups. (E) The concentrations of total bile acid in feces of control group, light group and lithocholic acid (LCA) group.

Through the analysis of metabolomics data and tryptophan metabolic pathway, we screened out six metabolites and carried out a 3‐week rescue experiment. It was found that all of them had different degrees of rescue effects, among which the two metabolites of LCA and NorDCA showed striking rescue effects. Figure 7A shows the representative images of MII oocytes, two‐cell embryos and blastocyst development in eleven repeat trials, and Figure 7B shows the degenerated oocyte rates of eleven repeat trials, in which the degenerated oocyte rates in 30 mg/kg LCA,15 mg/kg NorDCA and 30 mg/kg NorDCA were significantly lower than in the Light group (*p* < .05). The development rates of two‐cell embryos in 30 mg/kg LCA,15 mg/kg NorDCA, 30 mg/kg NorDCA,1 mg/kg AMK, and12.5ug/kg VD3 groups were significantly higher than in the Light group (*p* < .05, Figure 7C). The blastocyst development rate of each group was also significantly higher than that of the light group (*p* < .05, Figure 7D). It is worth noting that the blastocyst development rate of the 12.5ug/kg VD3 group reached 64.1%, and the blastocyst development rate of the 15 mg/kg NorDCA group was 57.4%. In addition, supplement of 30 mg/kg LCA could significantly improve the decreased total bile acid concentration in the intestine (*p* < .05, Figure 7E).

## 3 DISCUSSION

**FIGURE 8:**
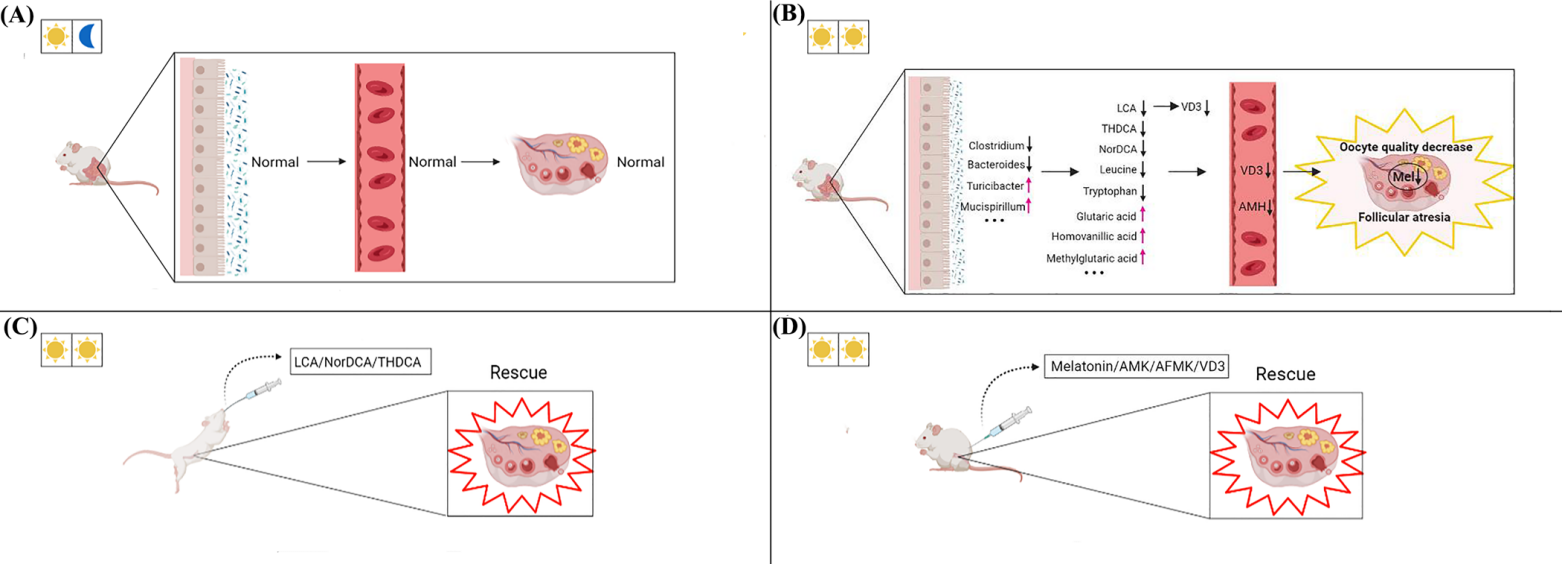
Graphical abstract. Diagram illustrating that vitamin D metabolism along the gut‐ovarian axis is essential for oocyte quality and embryonic development. The disrupted vitamin D absorption was affected by reduced levels of bile acids such as lithocholic acid (LCA), which was significantly associated with increased abundance of *Turicibacter*. The abnormal vitamin D metabolism affected the levels of AMH hormone in the blood and melatonin in the follicular fluid. This was a major cause for decreased oocyte quality and embryonic development, as confirmed by the injection of VD3. In addition, we identified a total of seven metabolites that were able to rescue quality and embryonic development of oocytes from mice exposed to continuous light.

Staying up late has become a way of life for more and more young people, and continuous light exposure leads to the decline of fertility, but the underlying mechanism is unknown. Therefore, it is of great significance to explore how continuous light exposure leads to the decline of oocyte quality and fertility as well as the potential of prevention and treatment by drugs. In this study, we showed that the estrous cycle of mice exposed to continuous light was prolonged, the concentrations of AMH hormone and melatonin in follicular fluid were decreased, while oocyte ROS level was increased, leading to decline of oocyte quality and embryonic development (Figure 8). Zhang et al. also found that continuous light exposure could hinder oocyte maturation and decrease the quality of oocytes, but the underlying mechanism is not clear.^15^ In this study, we conducted metabolomics profiling and 16S rDNA‐seq of mouse feces, and found that continuous light exposure altered metabolites that were mainly concentrated in the pathway of bile acid biosynthesis. The total bile acid concentration in the feces was significantly reduced. Continuous light exposure decreased the concentration of a variety of bile acids including LCA, NorDCA, THDCA, isoalloLCA, 7‐KetoLCA and CDCA, which was also verified by Elisa detection.

LCA is an agonist of vitamin D receptor,^16^ and we found that vitamin D concentrations in the light group decreased in feces and blood. Vitamin D is considered to be related to reproduction. Women with higher levels of vitamin D have a higher pregnancy rate, which confirms its important role in reproductive medicine.^17^, ^18^ Vitamin D was closely related to the level of AMH hormone. Merhi et al. found that vitamin D was proportional to AMH hormone in the serum.^19^ In addition, not enough serotonin was produced without adequate vitamin D.^20^ Serotonin was a precursor of melatonin, so melatonin production was limited.^21^ Therefore, the decrease of vitamin D concentration in the light group may be the reason for the decrease of AMH hormone and melatonin in follicular fluid. In addition, continuous light exposure led to an increase in the concentrations of three microbial metabolites of glutaric acid, homovanillic acid and methylglutaric acid that cause oxidative stress in the intestinal tract.^22^, ^23^, ^24^ After the three metabolites are absorbed into the blood, they would reach the ovary along with the blood circulation, causing oxidative stress in the ovary, increasing the ROS level of the oocyte, and eventually leading to the decline of oocyte quality. Vaccaro et al. found a similar phenomenon, with sleep deprivation increasing ROS level in the gut and ultimately leading to a significantly shorter lifespan in fruit flies.^25^ Abnormal hormone levels and excessive oxidative stress would cause follicular atresia, resulting in the decline of oocyte quality and embryonic developmental ability.

In addition, there are three points worthy of attention. First, isoalloLCA is a special secondary bile acid that can resist *Clostridioides difficile*, *Enterococcus faecium* and other infections, and the concentration of fecal samples was higher in centenarians.^26^ Continuous light exposure reduced the concentration of isoalloLCA, indicating that continuous light may reduce the lifespan of mice. Second, Harmon et al found that cancer cells could inhibit T cells that attack cancer cells by metabolizing lactic acid and maintaining a high lactic acid environment, thus protecting themselves from being attacked by the immune system. In addition, in colorectal cancer biopsy samples, a high level of lactic acid generally indicates a high risk of metastasis, poor survival and poor prognosis.^27^ Continuous light exposure increased the concentration of lactic acid in the gut (*p* < .05, online Figure S5A–D). Third, continuous light exposure reduces the concentrations of essential amino acids such as tryptophan, leucine and phenylalanine. As essential amino acids, they play an important role in maintaining cell proliferation and survival.^28^

Studies have reported that three bacteria including *Clostridium*, *Bacteroides* and *Parabacteroides* are involved in the process of converting primary bile acids to secondary bile acids. Our results of 16S rDNA‐seq showed that continuous light exposure caused the decline of the abundance of *Clostridium* and *Bacteroides*. Lefse analysis showed that *Clostridium* was one of the characteristic bacteria of the control group, which explained the decline of multiple secondary bile acids in the metabolomics profiling.^29^, ^30^ *Turicibacter* is pro‐inflammatory, causes oxidative stress, and leads to an increase in lactic acid.^31^ Continuous light exposure increased the abundance of *Turicibacter*, which explained increased oxidative stress and lactic acid in the metabolomics profiling. Moreover, the results of our Spearman's correlation analysis showed that the abundance of *Turicibacter* bacteria was significantly correlated with LCA, CDCA, NorDCA, 7‐KetoLCA, tryptophan and leucine. Kemis et al. and Thangamani et al. found an interaction between *Turicibacter* and bile acids.^32^, ^33^ In addition, continuous light exposure leads to increased abundance of *Mucispirillum*, which also causes oxidative stress.^34^

Melatonin is a hormone secreted by the pineal gland of the brain. It regulates the immune system, nervous system and reproductive system. Studies have confirmed that melatonin was the strongest known endogenous free radical scavenger and could promote mammalian oocyte maturation, follicular development and embryonic development.^35^, ^36^ In this study, we established a rescue approach with melatonin and found that melatonin significantly rescued the decline in oocyte quality and embryonic development caused by continuous light exposure. In terms of microbial metabolites, melatonin could significantly increase the concentration of LCA, isoalloLCA and significantly reduce the concentration of homovanillic acid and M∖methylglutaric acid. In terms of gut microbiota, melatonin could increase the abundance of *Parabacteroides* and decrease the abundance of *Turicibacter* and *Mucispirillum*, which explains the reason that melatonin could increase the concentration of secondary bile acid and reduce the concentration of metabolites causing oxidative stress. In addition, some studies have shown that the downstream metabolites AFMK and AMK of melatonin were more efficient in cleaning up oxidative stress than melatonin.^37^, ^38^

In order to further verify our experimental results and find more metabolites that could improve the quality of oocytes we screened out six metabolites and carried out a 3‐week rescue experiment: LCA, NorDCA, THDCA, AMK, AFMK and VD3. Because the active substance of vitamin D is VD3,^39^ we choose to inject VD3 to supplement the concentration of vitamin D. Compared with the data from the continuous light group, we mainly evaluated the saving effect of metabolites from three aspects: first, whether there was a significant reduction in the degenerated oocyte rate after superovulation; second, whether there was a significant decrease in oocyte mortality after parthenogenetic activation for 3 h (*p* < .05, online figure S6A,B); and third, whether the two‐cell embryos and blastocyst development were significantly increased. Excitedly, we found that all the six metabolites had varying degrees of rescue effects, among which 30 mg/kg LCA and 15 mg/kg NorDCA had the best rescue effect, and 30 mg/kg LCA could significantly increase the concentration of total bile acid in feces, which indicated that the decrease of secondary bile acid concentration was the main reason for the decline of oocyte quality caused by continuous light exposure. This is also the first time to find that LCA and NorDCA could significantly improve the quality of oocytes. At present, there are very few reports on NorDCA, which is worthy of our further study. It is worth noting that although 12.5 μg/kg VD3 injection did not significantly reduce the degenerated oocytes rate, it significantly increased the blastocyst development rate up to 64.1%. In addition, by comparing the results of high and low concentrations of three metabolites of NorDCA, AMK and VD3, it was found that the low concentration had better rescue effects, which suggested that we should give priority to the use of low concentrations when supplementing metabolites in the future.

In summary, through our rescue experiments, we found that the main reason for the decline of oocyte quality caused by continuous light exposure was the disorder of gut microbiota, and that the decrease of microbial metabolites such as secondary bile acids led to the decrease of vitamin D concentration, finally affected the level of AMH and melatonin in follicular fluid, resulting in the decline of oocyte quality. The secondary reason was that continuous light exposure leads to increased concentrations of metabolites that produce oxidative stress in the gut. After these metabolites are absorbed into the blood, ovarian oxidative stress occurs, and the ROS level increases in oocytes, ultimately leading to the decline of oocyte quality. Our findings of oocyte quality rescue by secondary bile acids LCA and NorDCA may have significant clinical value.

## CONFLICT OF INTEREST STATEMENT

The authors declare no conflict of interest.

## Supporting information

Supporting Information

Supporting Information

Supporting Information

Supporting Information

Supporting Information

Supporting Information

Supporting Information

## Data Availability

The 16S rDNA gene sequencing data is available in the SRA database repository https://www.ncbi.nlm.nih.gov/sra/PRJNA916844. All data can be obtained in this manuscript or from the authors upon request.
