# Supplementary material for: Gut microbiota–bile acid‐vitamin D axis plays an important role in determining oocyte quality and embryonic development
Source: Clin Transl Med. 2023 Oct 17;13(10):e1236. doi: 10.1002/ctm2.1236 (PMC10580005; doi:10.1002/ctm2.1236)
Supplement: Supplementary file 1 — Supporting Information [file CTM2-13-e1236-s005.docx]

**Supplemental Information**

This file includes:

Materials and Methods

Supplementary Figures 1 to 6 and figure legends

**Materials and Methods:**

**Animals**

All eight-week-old female ICR mice were purchased from SPF Biotechnology Co. Ltd. (Beijing) and acclimated for a week in a conventional animal facility with a 12 h light:12 h dark cycle (lights on 7:00 AM to 7:00 PM; light intensity: 250 lux) and an ambient temperature of 22 ± 2℃. The mice had free access to a chow diet and water. The experimental protocols and animal handling procedures were reviewed and approved by the Institutional Animal Care and Use Committee of Guangdong Second Provincial General Hospital.

**Establishment of continuous light model**

8-week-old female ICR mice were exposed to constant light (24h/day bright light; light intensity:250lux) for 8 weeks. The control group was exposed to 12h light: 12h dark cycle (light duration from 7:00 am to 7:00 pm, light intensity: 250lux).The estrus cycle was monitored daily for the last 6 days of the whole procedure by a cell shedding microscope. After the modeling, mature MII eggs were obtained by intraperitoneal injection of PMSG and HCG, and ovaries, blood and feces were collected for the following experiments.

**TUNEL assays**

Ovaries were collected, washed and fixed in formalin overnight. After gradient dehydration and paraffin embedding, they were sectioned at 5 µm thickness. One Step TUNEL Apoptosis Assay Kit (Beyotime, C1086) was used for TUNEL assay of the ovarian sections, according to the manufacturer's protocol.

**Immunofluorescence and scanning confocal microscopy**

To assess the ROS level of oocytes, the MII oocytes were incubated in M2 medium with 10 mM DCFH diacetate (Cat #S0033; Beyotime) at 37°C for 25 min, washing in M2 medium at least twice. They were measured at 488nm (green fluorescence) wavelength by using a confocal laser-scanning microscope.

To assess mitochondrial distribution, the MII oocytes were incubated with 200nm MitoTracker Red (molecular probe) in darkness at 37°C for 25 minutes, and washing in M2 medium at least twice. They were measured at 561nm (red fluorescence) wave length by using a confocal laser scanning microscope.

To assess spindle assembly, the MII oocytes were fixed in 4% paraformaldehyde phosphate-buffered saline (PBS) for 30 min at room temperature. The fixed oocytes were infiltrated with 0.5% Triton X-100 for 20min. After being blocked in PBS containing 1% BSA for 1h at room temperature, they were incubated with 1:800 anti-α-tubulin-FITC antibody at 4℃ overnight (Cat#2125; Beverly). Oocytes were washed and stained with Hoechst33342 for 15min.

**mtDNA content determination by quantitative real‐time PCR**

Real‐time PCR was performed to determine the total amount of mtDNA of each single oocyte in the three groups. Briefly, a single oocyte was loaded into a PCR tube with 10 µl lysis buffer (proteinase K added) and incubated at 55°C for 2 h and 95°C for 10 min and then the samples were used directly for quantitative PCR (qPCR) analysis. The mouse mtDNA‐specific primers: B6 forward, AACCTGGCACTGAGTCACCA, and B6 reverse, GGGTCTGAGTGTATATATCATGAAGAGAAT. To obtain the standard curve. The cycling conditions included an initial phase of 10 min at 95°C and 40 cycles of 15 s at 95°C and 1 min at 60°C. The melting temperature was 76.5°C. Linear regression analysis of all standard curves for samples with copy numbers between 10^2^ and 10^8^ showed a correlation coefficient higher than 0.98. All measurements were performed in triplicates.

**ELISA assay of total bile acid and vitamin D**

Follicular fluid was obtained from the ovary and melatonin concentration was analyzed by ELISA kit (RGB-60079M). The blood was transferred to a 4°C centrifuge for 20 min, and the serum was absorbed with a pipe gun, and the concentration analysis of AMH (RGB-60606M) and vitamin D (Cayman, 501050) was carried out by ELISA kit. The collected feces homogenate was analyzed by ELISA kit for concentration of total bile acid (TBA, RGB-40087), vitamin D (Cayman, 501050), IDO1(SBJ-M1100).

**Parthenogenetic activation and embryonic development**

Our pre‐experimentation determined the optimum concentration of SrCl_2_ and cytochalasin B (CB) for oocyte activation. Oocytes were placed into Ca^2+^‐free CZB medium supplemented with 5 mM SrCl_2_ (SrCl_2_, 10025 70‐4; Sangon Biotech, Shanghai, China) and 5 mg/ml CB for 3.5 hr to induce parthenogenetic activation simultaneously. Then oocytes were further cultured in conventional KSOM medium without CB, and the formation of two-cell embryos and blastocysts development was observed at 24 and 96 hr after start of culture, respectively.

**Sample preparation and analysis for metabolomics**

The quantification of microbial metabolites was performed through Metabo-Profile (Shanghai, China) using the UPLCMS/MS system. All target standards were obtained from Sigma-Aldrich (St. Louis, Missouri, USA) and TRC Chemical Company (Toronto, Canada). Fecal samples were processed using the previously published method of simply extracting metabolites by freeze-drying 5mg of fecal matter with zirconia beads, 25 µl of deionized water, and 120 µl of methanol containing the inner target. The resulting supernatants were subjected to derivatization with 3-nitrophe-nylhydrazine(3-NPH) and N-(3-(dimethylamino)propyl)-N′-ethyl car-bodiimide (EDC)⋅HCl (Sigma-Aldrich, St. Louis, MO, USA). Subsequently, the derivatized samples were analyzed by ultra- performance liquid chromatography coupled to a tandem mass spectrometry (UPLC-MS/MS) system (ACQUITY UPLC-Xevo TQ-S, Waters Corp., Milford, MA, USA). All of the standards were obtained from Sigma-Aldrich (St. Louis, MO, USA), Steraloids Inc. (Newport, RI, USA) and TRC Chemicals (Toronto, ON, Canada).

**Gut microbiota analysis**

Genomic DNA was extracted using the OMEGA Soil DNA Kit (Omega Bio-Tek, Norcross, GA, USA), following the manufacturer’s instructions. The V3-V4 region of bacterial 16S rRNA gene was amplified with forward primer 338F (5'-ACTCCTACGGGAGGCAGCA-3') and reverse primer 806R (5'-GGACTACHVGGGTWTCTAAT-3'). The amplified products were purified with Vazyme VAHTSTM DNA Clean Beads (Vazyme, Nanjing, China), and quantified with Quant-it PicoGreen dsDNA Assay Kit (Invitgen, Carlsad, CA, USA). Illumina NovaSeq platform was used for sequencing. The original sequence data was demultiplexed to cut the primers. The DADA2 plugin was then used for quality filtering, denoising, merging, and chimera removal of the sequences. Microbiome bioinformatics mainly used QIIME2 and R packages to calculate and visualize the classification and heat map of differential abundance. In addition to log10LDA>2.0, we also used linear discriminant analysis effect size to detect differential abundance of genera in the three groups.

**Rescue experiments**

We designed a total of seven metabolites rescue experiments. This could be divided into two categories according to different administration methods. One was the secondary bile acids that need gavage, including LCA (Yuan Ye, B28100), NorDCA (TRC, N672000) and THDCA (Yuan Ye, S31796). The other was metabolites that require intraperitoneal injection, including melatonin (Sigma, M5250), AMK (TRC, A186500), AFMK (TRC,A176100), and VD3 (Sigma, C9756). Except for melatonin with a rescue cycle of 8 weeks, all the other metabolites had a 3 weeks rescue cycle. Combined with the published literature, the concentration of LCA was 30mg/kg. The concentrations of NorDCA were 30mg/kg and 15mg/kg, respectively. The concentration of THDCA was 30mg/kg. The concentration of AFMK was 1mg/kg; The concentrations of AMK were 1mg/kg and 2mg/kg. The concentrations of VD3 were 12.5 µg/kg and 25µg/kg.

**Statistical analysis**

We used the GraphPad Prism (USA) for statistical analysis. The bioassay assay results were presented in the form of means ± SEM. All results were considered statistically significant at *P*<0.05. Statistical significance between two groups was determined by Student’s t test. Univariate analysis of variance (ANOVA) and Tukey's multiple comparison test were used to assess the statistical significance of differences between three or more groups. Differential metabolites were defined as those with variable importance in the projection (VIP) >1.0 and adjusted *P* value less than 0.05.

**Supplementary figure legends**

Supplementary Figure 1. Constant light exposure caused an increase in the concentration of the enzyme IDO1 in the feces. (A) The metabolic pathway diagram of tryptophan. (B) The concentration of the tryptophan rate-limiting enzyme IDO1.

Supplementary Figure 2. Continuous light exposure resulted in significant abnormalities in the concentrations of seven metabolites, all of which were improved after melatonin supplementation. (A) Continuous light exposure caused abnormal concentration of metabolites such as lactic acid, aspartic acid and glutamine. (B) Continuous light exposure caused significantly higher concentration of lactic acid and lower concentration of aspartic acid and glutamine. (C) The abnormal contents of indoleacetic acid, benzoic acid, hydroxyphenyl lactic acid and citramalic acid were induced by persistent light.

Supplementary Figure 3. Important markers of differences among the three groups. (A) The abundance of *Bacteroides* decreased in the Light group and the Mel group. (B)

The abundance of *Clostridium* decreased in the Light group and the Mel group. (C) The abundance of *Parabacteroides* decreased in the Light group, but increased in the Control group and Mel group. (D) The abundance of *Turicibacter* increased in the Light group, but decreased in the Control group and Mel group. (E) The abundance of *Sphingomonas* increased in the Light group, but decreased in the Control group and Mel group. (F) The abundance of *Lactobacillus* significantly increased in the Mel group. (G) The abundance of *Roseburia* increased in the Light group, but decreased in the Control group and Mel group. (H) The abundance of *Oscillospira* increased in the Light group, but decreased in the Control group and Mel group. (I) The abundance of *Coprococcus* increased in the Light group, but decreased in the Control group and Mel group. (J) The abundance of *Mucispirillum* increased in the Light group, but decreased in the Control group and Mel group. (K) The abundance of *Odoribacter* increased in the Light group, but decreased in the Control group and Mel group. (L) The abundance of *Alistipes* increased in the Light group, but decreased in the Control group and Mel group.

Supplementary Figure 4. Spearman’s correlation analysis of differential microbial metabolites with gut microbiota in the Light group. (A) Spearman’s correlation analysis of eight gut microbiota levels and different microbial metabolites levels. (B-G) *Sphingomonas* was positively correlated with glutaric acid, homovanillic acid, methylgutaric acid, lactic acid, phenyllactic acid, and indoleacetic acid, respectively.

Supplementary Figure 5. Comparison of fecal VD3 levels and serum VD3 levels between the Light and Control groups. (A) Compared with the control group, fecal VD3 concentration in the Light group was significantly decreased, p=0.0005. (B) Compared with the control group, fecal VD3 concentration in the Light group was significantly decreased, p＜0.0001.

Supplementary Figure 6. After 3 hr of parthenogenetic activation, oocyte survival rates of eight groups were analyzed. (A) Diagram of oocyte survival in eight groups after 3 hr of parthenogenetic activation. (B) After 3 hr of parthenogenetic activation, oocyte survival rates in the other seven groups were significantly higher than that in the Light group.
