# Supplementary figures and images for: Gut microbiota–bile acid‐vitamin D axis plays an important role in determining oocyte quality and embryonic development
Source: Clin Transl Med. 2023 Oct 17;13(10):e1236. doi: 10.1002/ctm2.1236 (PMC10580005; doi:10.1002/ctm2.1236)

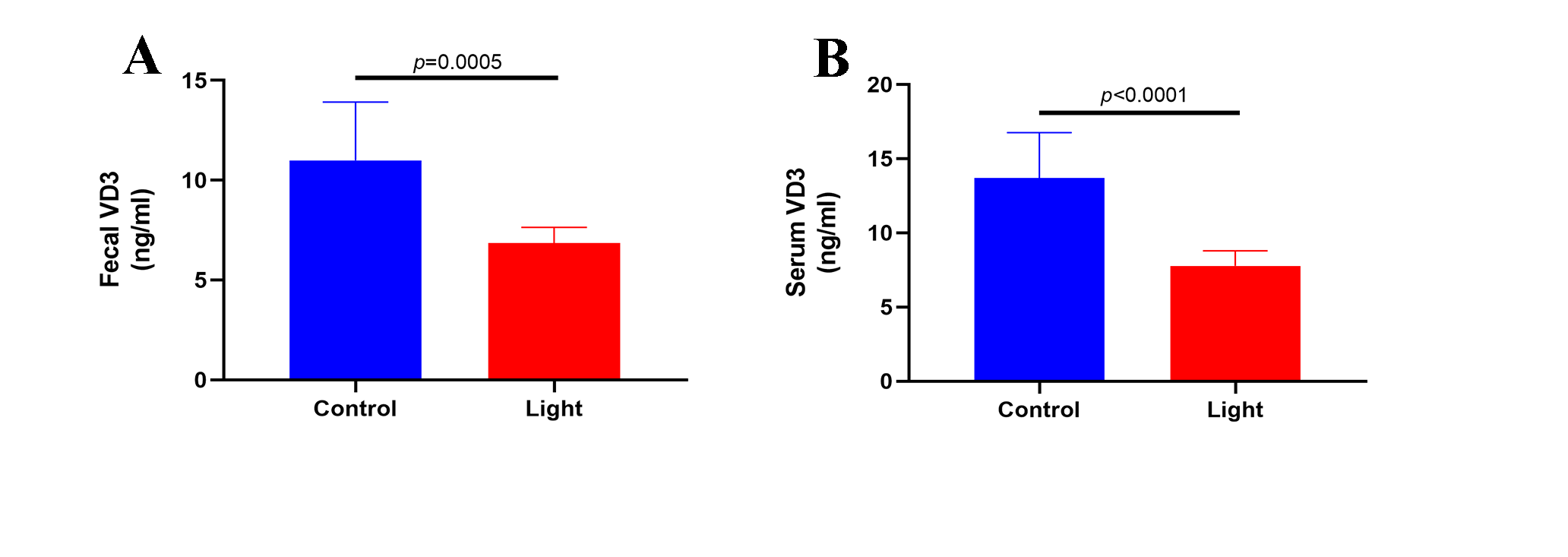

Supplement: Supplementary file 2 — Supporting Information [file CTM2-13-e1236-s004.tif]

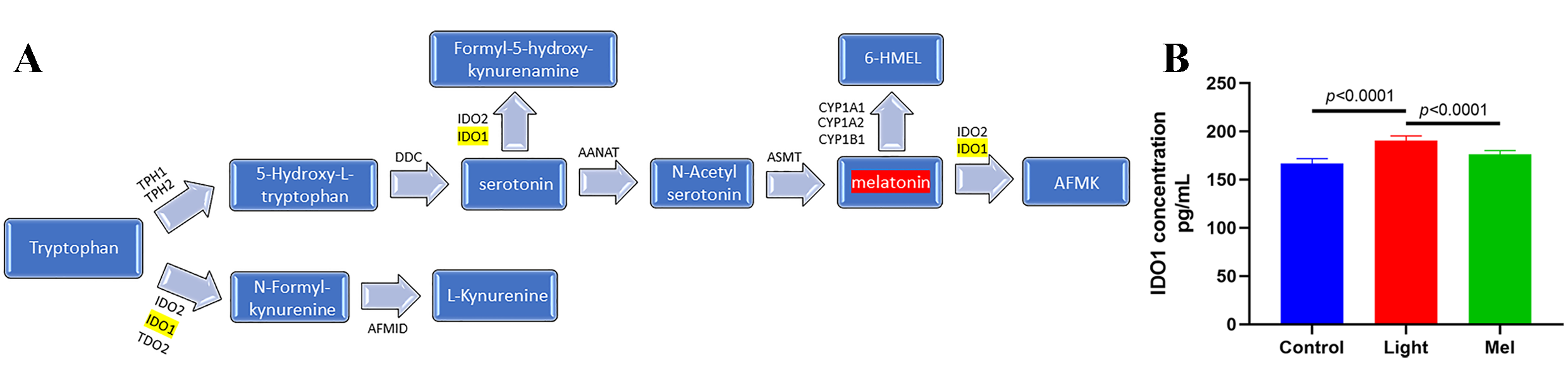

Supplement: Supplementary file 3 — Supporting Information [file CTM2-13-e1236-s003.tif]

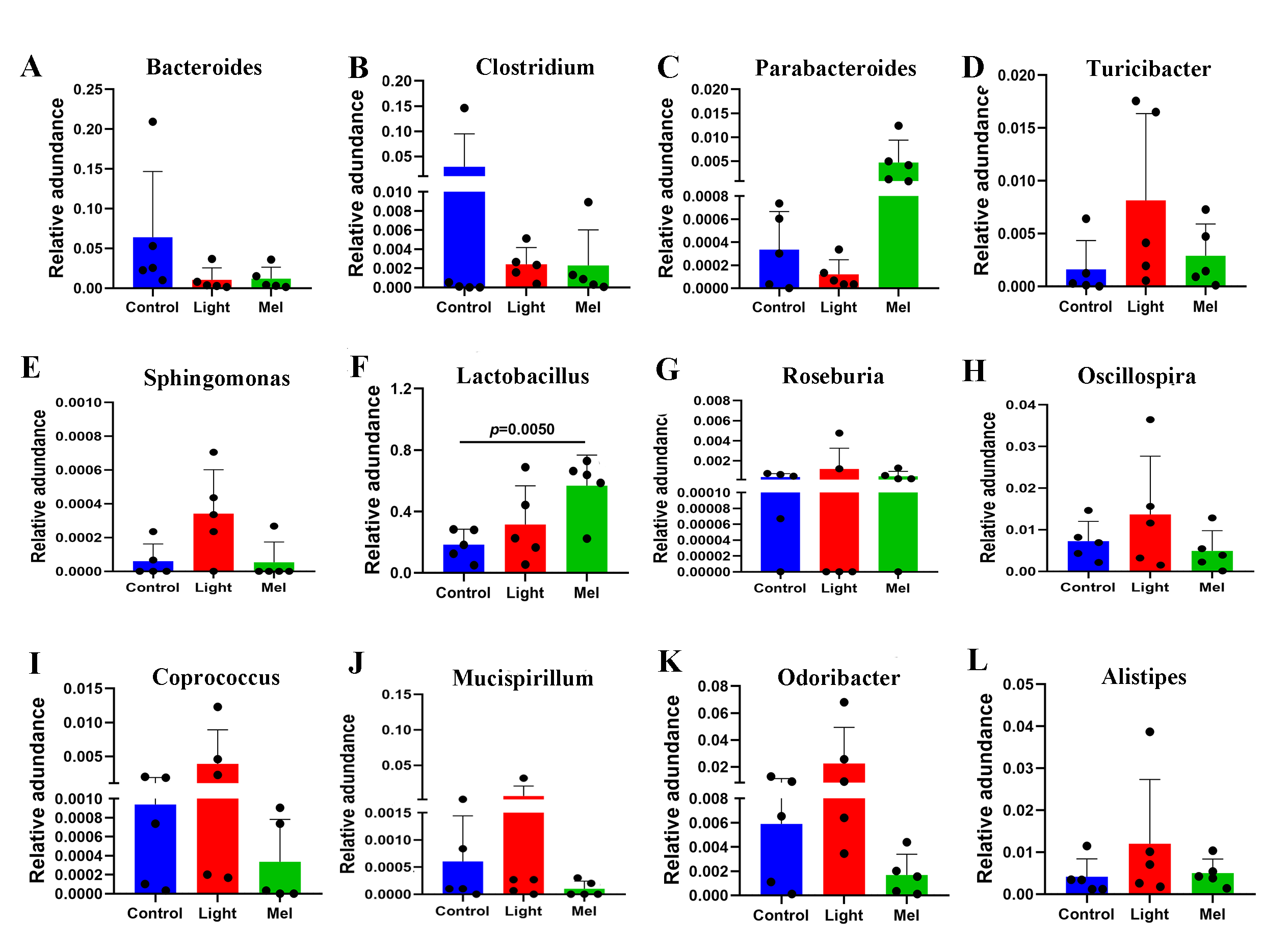

Supplement: Supplementary file 4 — Supporting Information [file CTM2-13-e1236-s001.tif]

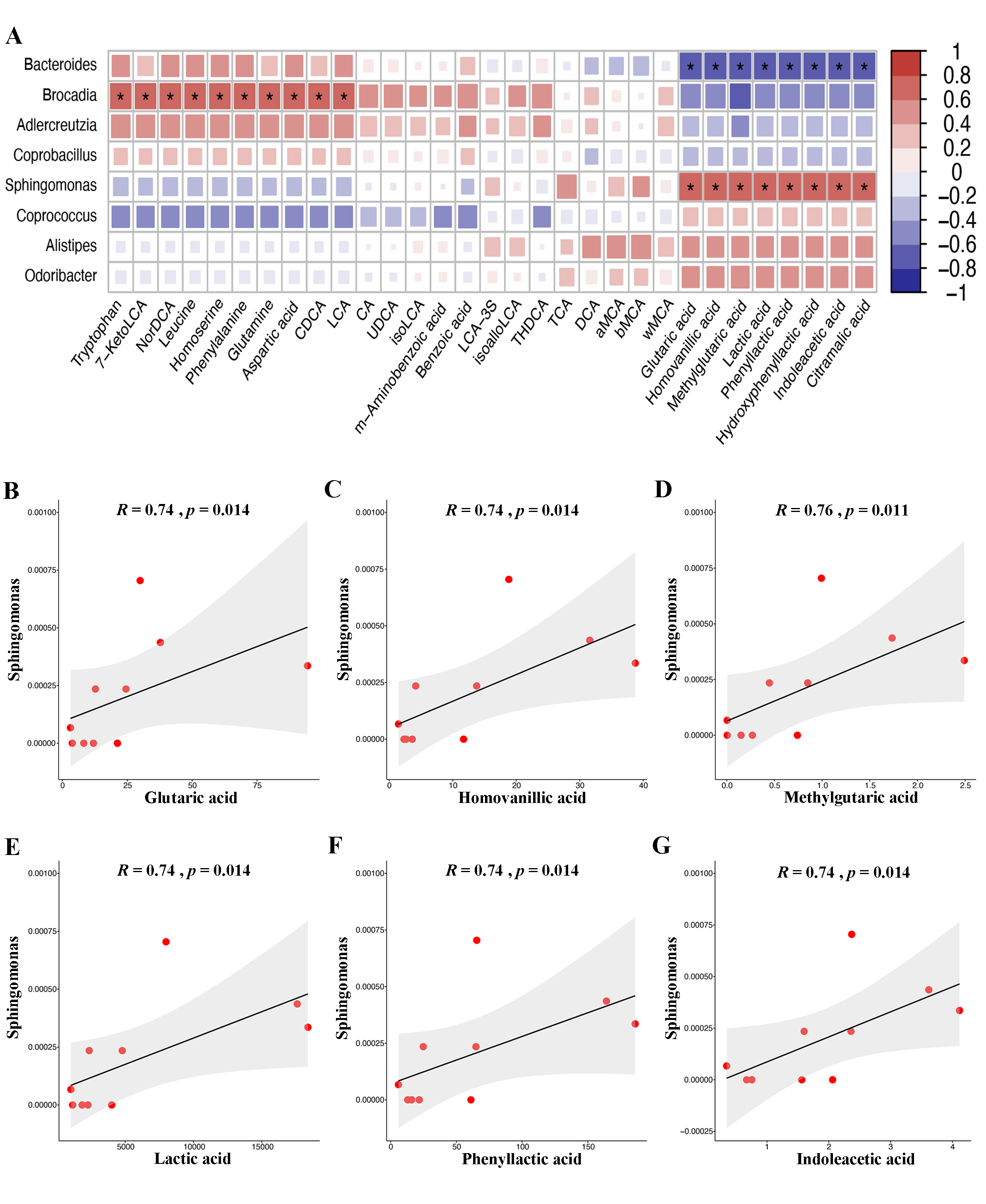

Supplement: Supplementary file 5 — Supporting Information [file CTM2-13-e1236-s006.tif]

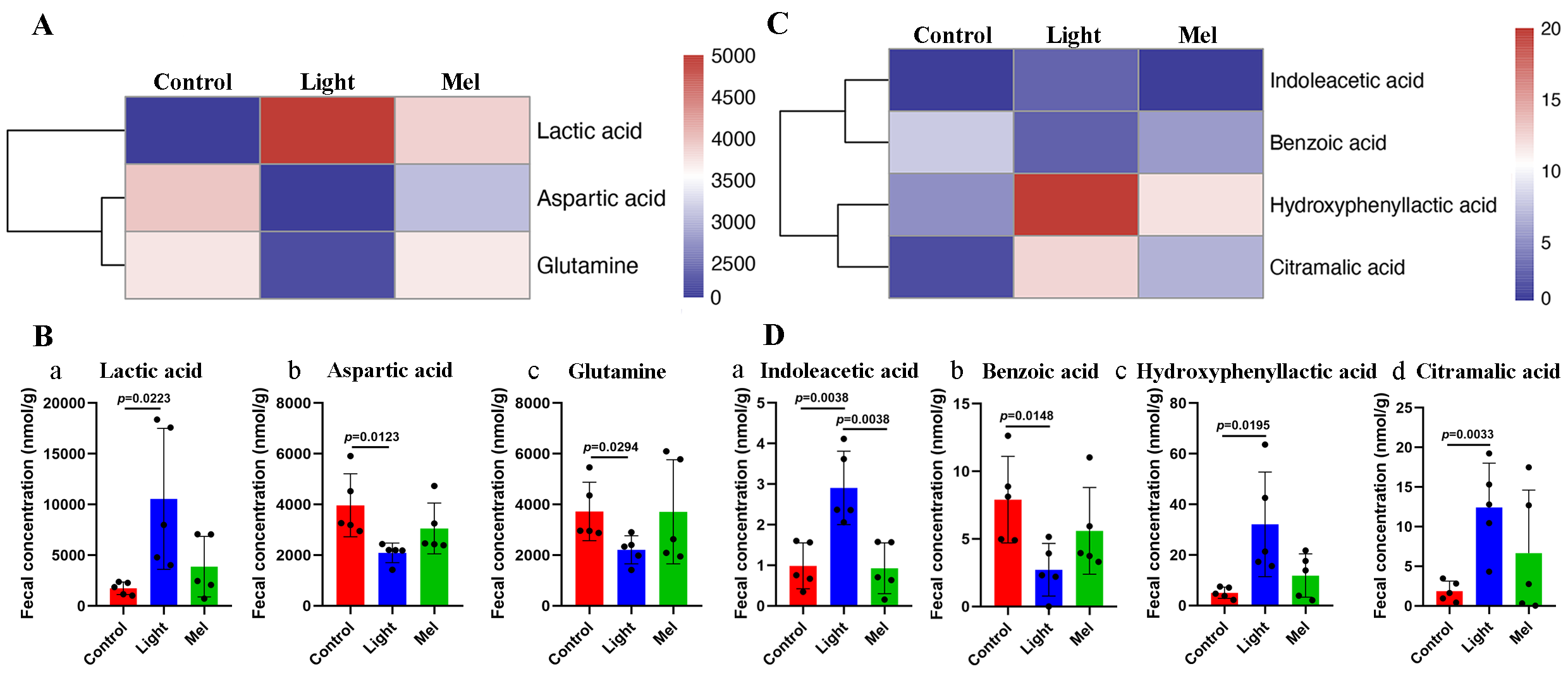

Supplement: Supplementary file 6 — Supporting Information [file CTM2-13-e1236-s002.tif]

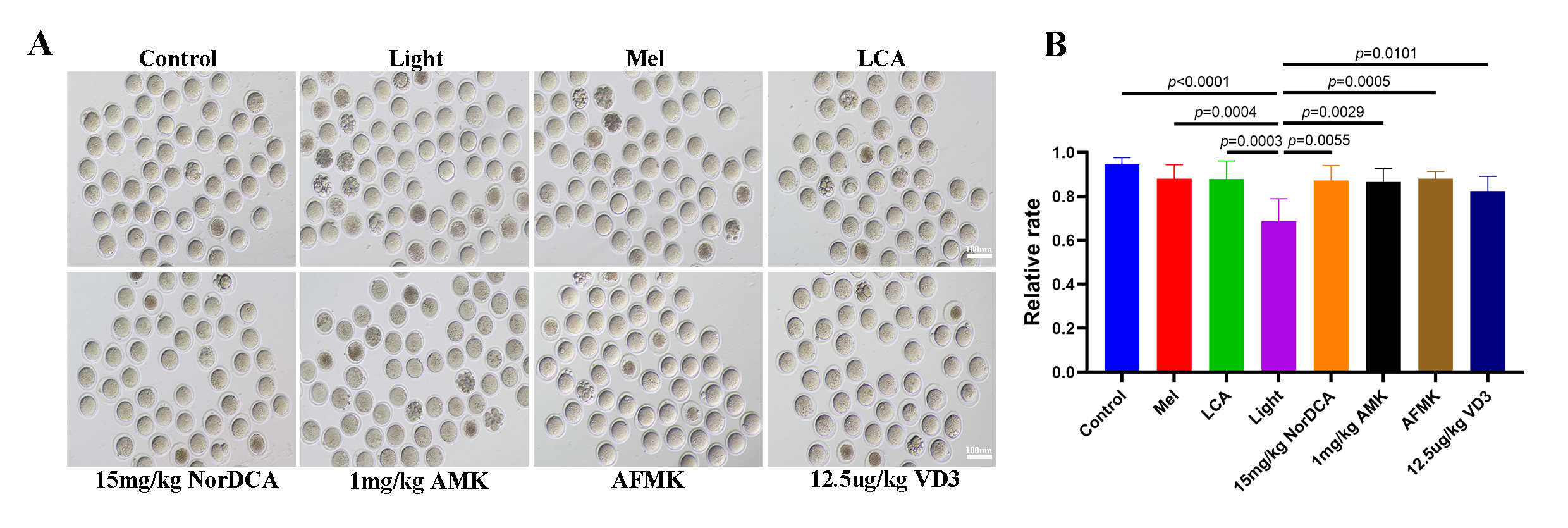

Supplement: Supplementary file 7 — Supporting Information [file CTM2-13-e1236-s007.tif]
